# Understanding Flavivirus Capsid Protein Functions: The Tip of the Iceberg

**DOI:** 10.3390/pathogens9010042

**Published:** 2020-01-05

**Authors:** Stephanea Sotcheff, Andrew Routh

**Affiliations:** 1 Department of Biochemistry and Molecular Biology, The University of Texas Medical Branch, Galveston, TX 77550, USA; slsotche@utmb.edu; 2 Sealy Center for Structural Biology and Molecular Biophysics, The University of Texas Medical Branch, Galveston, TX 77550, USA

**Keywords:** flavivirus, capsid protein, antivirals, vaccines

## Abstract

Flaviviruses are enveloped positive-sense single-stranded RNA arboviruses, infectious to humans and many other animals and are transmitted primarily via tick or mosquito vectors. Capsid is the primary structural protein to interact with viral genome within virus particles and is therefore necessary for efficient packaging. However, in cells, capsid interacts with many proteins and nucleic acids and we are only beginning to understand the broad range of functions of flaviviral capsids. It is known that capsid dimers interact with the membrane of lipid droplets, aiding in both viral packaging and storage of capsid prior to packaging. However, capsid dimers can bind a range of nucleic acid templates *in vitro*, and likely interact with a range of targets during the flavivirus lifecycle. Capsid may interact with host RNAs, resulting in altered RNA splicing and RNA transcription. Capsid may also bind short interfering-RNAs and has been proposed to sequester these species to protect flaviviruses from the invertebrate siRNA pathways. Capsid can also be found in the nucleolus, where it wreaks havoc on ribosome biogenesis. Here we review flavivirus capsid structure, nucleic acid interactions and how these give rise to multiple functions. We also discuss how these features might be exploited either in the design of effective antivirals or novel vaccine strategies.

## 1. Introduction

Flaviviruses are arthropod-borne viruses that plague both tropic and sub-tropic regions. These viruses belong to the family *Flaviviridae* and genus *Flavivirus*. There are slightly over 70 species of flaviviruses that have been discovered so far [1]. Of these, roughly half are mosquito-borne, including the heavily studied: yellow fever (YFV), West Nile (WNV), dengue (DENV), Japanese encephalitis (JEV), and Zika (ZIKV) viruses, which will be the focus of this review [2]. These viruses appear to affect tropic and sub-tropic regions but pose a serious global health risk due to geographic expansion of mosquito vectors [3,4,5].Only a small subset of infections result in symptoms, ranging from mild fever to hemorrhagic fever or encephalitis to potentially death. The 2015–2016 outbreak of ZIKV in South America was also associated with microcephaly in infants and the development of Guillain-Barre syndrome in adults [2,6]. There are only a few Food and Drug Administration (FDA) approved vaccines for humans currently available for a few mosquito-borne flaviviruses (namely YFV, JEV, and DENV). However, the DENV vaccine has shown limited efficacy against all DENV serotypes [7,8] and resulted in injury to children in the Philippines, causing safety concerns [9]. Even with developments in the design of safe and efficacious vaccines, there are unfortunately no anti-viral treatments clinically available for infected individuals. As the features of the life cycle appear to be conserved across flaviviruses, there has been much work done to identify pan-flaviviral anti-viral targets and to engineer drugs to halt pathogenesis at various stages [1]. Thus, it is important to work towards a greater understanding of the molecular mechanisms throughout the viral life cycle.

**Figure 1:**
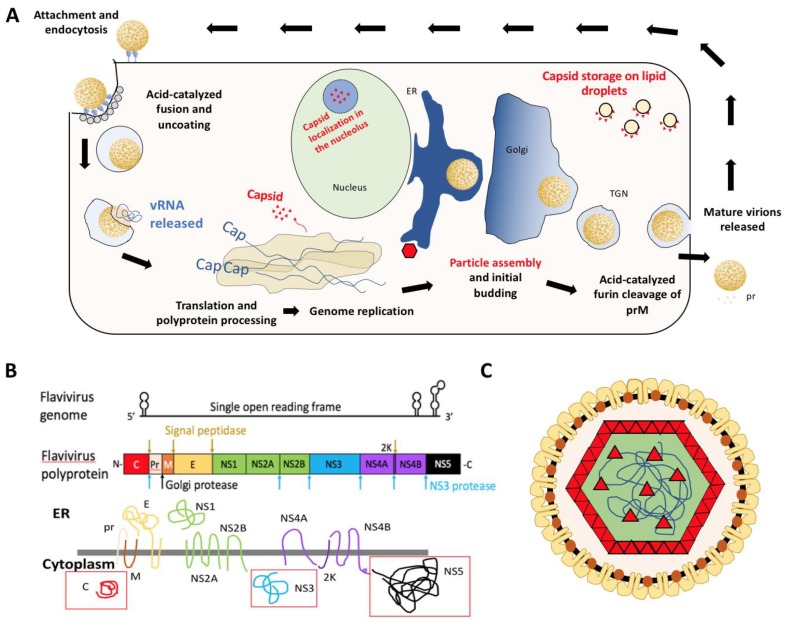
(**A**) Diagram of flavivirus life cycle with emphasis on distribution of viral capsid (red). (**B**) Schematic of the flaviviral genome, polyprotein, and transmembrane viral proteins. Adapted from Ming et al. [3]. Red boxes indicate soluble viral proteins in the cytoplasm. The primary focus of this review will be the viral capsid [C] protein. (**C**) Diagram of flavivirus particle with E (yellow), M (orange), and C (red) proteins. E and M proteins span the membrane derived from host endoplasmic reticulum and capsid interacts with these proteins as well as coats the viral genome (blue line).

Flaviviruses are enveloped positive sense single-stranded RNA (+ssRNA) viruses that package their ~11 kb genome into individual virus particles that are approximately 50 nm in diameter [2]. The virions enter the cell via receptor-mediated endocytosis and fuse with the endosomal membrane, releasing viral nucleocapsid into the cytoplasm [1]. Uncoating is complete when the genome is released from the capsid proteins. The viral genome contains a single open reading frame that must be translated at the endoplasmic reticulum (ER) membrane as a viral polyprotein [6,10,11] to generate the viral proteins including the RNA-dependent RNA polymerase (RdRP) required for genome replication (Figure 1A). Thereafter, the positive sense genome can be either used to generate a negative-sense template or for translation. Both during and after translation into the ER membrane, the polyprotein is processed to produce 10 viral proteins: three structural (capsid: C, pre-membrane: prM, and envelope: E), as well as seven non-structural proteins (NS1, NS2A, NS2B, NS3, NS4A, NS4B, and NS5). This yields three soluble viral proteins in the cytoplasm: C, NS3 (protease/helicase), and NS5 (RdRP with methyl-transferase activity) as indicated by red boxes in Figure 1B. Following synthesis of nascent positive-sense genome in the cytoplasm, the RNA is encapsidated into nucleocapsid particles which begin budding into the ER lumen. These particles traverse through the secretory pathway, undergoing furin-mediated cleavage of prM to produce mature virus particles that are expelled from the cell via exocytosis (Figure 1) [6,10].

The structural proteins are necessary for the formation of virus particles. Of these proteins, (pr)M and E are integral membrane proteins, with E protruding on the particle surface. This protein is the primary antigen associated with recognition by neutralizing antibodies [12,13]. The protein of interest in this review, capsid, interacts with the viral genomic RNA within virions. This is the primary function of capsid protein. Prior to encapsidation, and discrete from genome replication, capsid dimers are stored on lipid droplets. Their ability to interact with lipid droplets is essential for efficient production of virus particles [14,15,16,17]. Capsid is also able to enter the nucleus and wreak havoc on ribosome biogenesis and the host transcriptome [18,19,20,21,22,23]. The function of these interactions within the nucleus and nucleolus in the context of the flavivirus life cycle is poorly understood. While the non-structural proteins are necessary for viral replication, virion assembly, and evasion of immune response, capsid appears to have additional roles aside from genome encapsidation as we will highlight throughout this review.

As translation, replication, and packaging are all distinct processes of the viral life cycle, they are separated spatiotemporally into compartments generated by the rearrangement of the ER membrane [11]. The rearrangement results in invaginations into the ER, which resemble vesicles with a pore connecting the interior to the cytoplasm. This creates a replication-favorable environment for the virus and has been viewed using 3D-electron tomography (ET), transmission and scanning electron microscopy (TEM and SEM, respectively) in both mammalian and insect cells [24,25,26,27]. Similar compartments have been seen for alpha- and nodaviruses [11]. These compartments have been referred to as “replication factories” and are roughly 60 to 90 nm in diameter, depending on cell type [27]. Within these replication factories three viral proteins are known to interact with the primarily double-stranded viral RNA: NS3, NS5, and capsid. Specific mutations in NS2A appear to hinder packaging [28,29]. It has recently been shown that dengue and ZIKV NS2A recruits the viral genome by binding specifically to the highly-structured 3′ UTR and the C-prM-E complex and protease to site of virion assembly coordinating capsid loading and subsequent virion assembly [30,31]. The NS3 helicase separates nascent (+) strand from template (−) strand starting at the 3′ end [32] and NS5 binds the 5′ UTR of the (+) sense viral genome and translocates to the 3′ end upon cyclization of the RNA to begin genome replication [33,34].

Capsid is a small ~12-kDa protein comprising the first ~105 residues of the flavivirus polyprotein. Capsid proteins both have a hydrophobic face that interacts with ER membrane as well as a basic face that interacts with viral RNA. Binding of RNA to capsid initiates particle formation by causing an aggregation of capsid. The aggregation of membrane-associated capsid into the nucleocapsid structure induces budding into the ER and the formation of immature virus particles. Capsid protein has been shown to bind multiple nucleic acid templates in a sequence-independent manner via electrostatic interactions with the negatively charged phosphate backbone [15,34,35]. The coupling of replication and packaging within these ER membrane compartments prevents capsid from packaging host RNAs. Until recently, it was unclear how capsid within these replication factories interacts specifically with the (+) sense viral genome, as there is some amount of (−) sense template available within these compartments. It was proposed that the (−) sense associated with nascent (+) sense intermediate prevents capsid binding [34,35]—however, this has not been definitively demonstrated. Recent studies demonstrating NS2A binding to 3′ UTR of genomic RNA and the subsequent localization to membrane-bound assembly factories suggest that this viral protein may nucleate or ‘seed’ the loading of capsid, thus providing the specificity in packaging of just the genomic RNA [31].

It has also been shown that capsid proteins co-localize with the nucleoli and lipid droplets within infected cells [17,35,36]. The capsid protein’s ability to leave the replication factories is interesting, and coupled with their ability to bind various nucleic acids (and host proteins) [35,36], suggests that flavivirus capsid may have multiple evolved functions beyond viral packaging. The result of these interactions can result in either the activation or repression of various pathways with deleterious effects, including apoptosis or cell cycle arrest [19,37]. In addition, host transcriptome-wide profiles have been generated for various flaviviral infections in different cell types, indicating broad gene-level changes [38,39,40]. As capsid is one of the few viral proteins released from the ER membrane and has been shown to leave the replication compartments and enter the nucleus, it is reasonable to consider that it may be at least partially responsible for changes to the host transcriptome. However, the specific protein or nucleic acid interactions and which pathways they affect that may result in these changes are not well understood.

Here, we emphasize the importance of the capsid in pathogenesis of mosquito-borne flaviviruses, from its role in genome packaging to alternative functions that are emerging. Although flavivirus genome structure and replication strategies are largely the same, this review focuses on the mosquito-borne flaviviruses. These viruses are more globally distributed than that of the tick-borne viruses such as tick-borne encephalitis virus [2,41], and crystal structures for capsid have been solved for ZIKV, JEV, and WNV [35,42,43]. The DENV C structure is similar to that of the other flavivirus capsids, as determined by nuclear magnetic resonance (NMR) [44]. Capsid’s various interactions throughout the host cell provide a larger scope of targets than the other flavivirus structural proteins. A greater understanding of these interactions and their implications should lead to insightful drug and vaccine design.

## 2. Structure of Flavivirus Capsid and Its Role in Packaging

**Figure 2:**
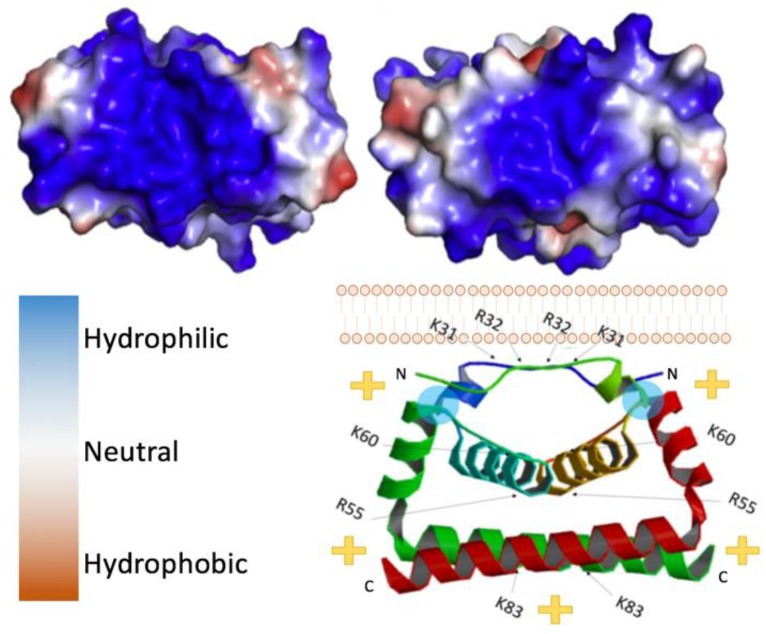
Flavivirus capsid structure. (Top left) Zika virus (ZIKV) C bottom view, 5YGH.pdb. (Top right) West Nile virus (WNV) C bottom view. Color key provided bottom left, 1SFK.pdb. (Bottom right) side view of ZIKV C dimer with its orientation to the lipid bilayer, indicating the polarity of the complex. Blue circles indicate location of residues necessary for interaction with the lipid membrane (L50 and L54).

Capsid proteins are the least genetically conserved of flavivirus proteins, but their structure and charge distribution are well conserved. Flavivirus capsids are highly charged proteins that contain multiple α-helical domains [44]. In its immature state, flavivirus capsid has a hydrophobic C-terminal domain (known as anchorC) that embeds in the ER membrane and is cleaved by NS3/2B protease releasing a soluble mature capsid, 99 to 114 residues in size [45]. Soluble capsid proteins readily dimerize *in vitro*, as confirmed by crosslinking studies [34]. Visualization of the dimers, although limited, has been found in asymmetric reconstructions of cryo-EM data, indicating that the dimer likely reflects the physiological conformation and is not an artifact of crystallization conditions. Initially, the three-dimensional structures of DENV and WNV capsid were solved using NMR and crystallography respectively [42,46]. At present, residues ~20–98 of many flavivirus capsids have been resolved in crystal structures (Figure 2) [35]. Each monomer is comprised of three to four alpha-helices, the first being the most flexible, consistently forming a right-handed bundle with the second helix (and third if there are four in total). Within dimers, these helices interact via hydrophobic interactions as illustrated in Figure 2. As depicted, the top (α1-α1′), bottom (α4-α4′), and core (α2-α2′) helices interact via hydrophobic interactions [35]. In contrast, the first ~20 residues are intrinsically disordered in solution and, similar to the final helix which extends away from the monomer core, are highly basic [17,45,46]. In contrast, the regions connecting α1-α2 and α1′-α2′ are relatively hydrophobic, allowing interactions with lipid bilayers [17]. In reference to Figure 2, where this hydrophobic linker-region is labeled by a cyan circle, the charge distribution places the basic residues on the “bottom” of the dimer, leaving the “top” of the dimer relatively uncharged.

Packaging of the viral genome into virus particles requires flavivirus capsid to interact with both the ER membrane (with prM and E proteins present) and with the genome. In addition to the disordered basic tails of flaviviral capsid, of interest is the large hydrophilic region on the bottom surface of each flavivirus capsid protein comprised of C-terminal domains of the dimer [47]. This face consists primarily of basic amino acids, lysine (K) and arginine (R). As shown by others, capsid appears to indiscriminately bind a range of nucleic acids *in vitro* including either single or double stranded RNA or DNA, suggesting non-specific interactions with the phosphate backbone [15,48,49]. However, the N-terminal domain of flavivirus capsids, unresolvable by crystallography, is flexible and also highly positively charged. Disordered, basic peptide tails are highly common in nucleic-acid binding proteins with roles in RNA or DNA compaction or packaging such as the lysine-rich eukaryotic histone tails [50] or arginine-rich motifs in viral capsid proteins from icosahedral non-enveloped viruses [51]. Similarly, in flavivirus capsid, the disordered basic tail is implicated in the packaging of viral genome. Alanine scanning of the conserved basic regions within the N-terminal domain (first 18 residues) of the DENV capsid resulted in very slow propagation of virus due to the release of fewer virus particles compared to wild-type [45]. Interestingly, these regions only needed be basic, and were not amino acid sequence specific. Thus, although the sequences of the N-terminus are not precisely conserved among flaviviruses, the two positively charged motifs/regions are.

Opposite the hydrophilic or basic face of the capsid dimer is a relatively hydrophobic surface, which is thought to interact with membranes and lipid droplets aiding in the assembly of flavivirus particles [15,17,35]. Lipid droplets are derived from the ER, housing neutral lipids in a phospholipid monolayer [52]. Notably, flavivirus infection results in an increase in lipid droplet production in various cell types [17]. Mutations to select hydrophobic residues on the hydrophobic surface of capsid can prevent its association with lipid droplets and attenuate virus. For example, L50 and L54 residues in the α2 helices of DENV C dimers are necessary for the capsid protein’s association with lipid droplets (location indicated by blue circles in the bottom portion of Figure 2). Mutations in these residues prevented this interaction with lipid droplets but also resulted in the production of fewer virus particles without affecting replication of the viral genome or translation of any viral proteins [17]. It appears that capsid proteins are stored on lipid droplets in the cytoplasm and mobilized for RNA packaging when needed, as lipids are constantly shuffled between the ER membrane and lipid droplets.

## 3. Alternative Functions of Flavivirus Capsids

Various groups have shown that, in addition to its association with lipid droplets, flavivirus capsids localize in the nucleoli [36,53,54]. The basic regions of the final helix of capsid protein appear to serve as a bi-partite nuclear localization sequence (NLS), identified by importin-α [19,20,36,53]. Importin-α binds these sequences in the cytoplasm and is then recognized by importin-β, which mediates transport of flavivirus capsid into the nucleus. However, across the mosquito-borne flavivirus capsids from different viruses there appear to be differing additional NLSs [45] which may use alternative pathways to enter the nucleus. Considering capsid’s role in viral packaging and the replication factories described above, it is interesting that capsid can dissociate with the phospholipid membranes of the ER and lipid droplets. This localization has indicated that capsid may have other functions or additional roles in flavivirus pathogenesis aside from packaging.

What is currently known of some of these alternative functions is that they can be either pro- or anti-viral. The presence of ZIKV C in nucleoli has been associated with ribosomal stress and an increase in programed cell death, especially in neuro-progenitor cells [6,20]. The localization of capsid in the nucleolus is not specific to ZIKV as it has been shown for DENV, WNV, and JEV as well [53,55]. A closer look into the effects of JEV C and DENV C in the nucleolus indicated interactions with ribosome biogenesis factors such as B23 and NPM1 or nucleolin, respectively [21,56]. WNV capsid appears to sequester p53 inhibitor MDM2/HDM2 in the nucleolus, allowing p53 to mediate apoptosis [23]. Mitochondrial membrane disruption may be due to p53-dependent up-regulation of Bax [23,55], ultimately resulting in the cleavage of pro-caspase 9 and apoptosis as well as local inflammation *in vivo* [37]. Prevention of ribosome biogenesis or cell death induced by flavivirus capsid could be considered anti-viral. However, it has also been shown that YFV C has the ability to suppress the anti-viral RNA silencing process in mosquitos by protecting viral dsRNA from processing via dicer [48]. This serves to allow propagation of the virus, however this method of vector immune response evasion does not appear to apply to all mosquito-borne flaviviruses [57]. Host protein Jab1 has also been shown to aid in the removal of WNV C from the nucleolus and present the viral protein to the proteasome for degradation, effectively preventing apoptosis in human lung carcinoma cell lines such as H1299 [19]. WNV C has also been shown to up-regulate protein phosphatase 2A, preventing the downstream upregulation of type I interferon genes [55]. Clearly, there are many interactions that capsid makes within cells that can either serve to aid or attenuate viral infection. We have only begun to scratch the surface of what these interactions are and how they affect host cells.

It has also been noted that flavivirus infection results in massive changes to the host/vector transcriptomes [38,39,40]. This may be the result of regulation of transcription, alternative splicing, or decay of transcripts. In the case of DENV serotype 1 in Huh7 cells, the largest changes observed were in differential isoforms, indicating alternative splicing [38]. However, ZIKV C has been shown to modulate the non-sense mediated mRNA decay (NMD) pathway [58]. Ingenuity pathway analysis (IPA) indicates that pathways associated with viral pathogenesis, protein synthesis, lipid and ceramide metabolism, and cell growth and proliferation are all up-regulated in response to flavivirus infection [38,39]. Generally, studies have focused on changes in the transcriptome in response to viral infection, but it may be interesting to see if introduction to individual viral proteins result in their own signatures.

Affinity purification and mass spectrometry determined that ZIKV C interacts with various NMD proteins, including poly-A binding protein C1 (PABPC1) and up-frameshift protein 1 (UPF1) [58]. This provides some insight into the global transcriptome changes, but there has also been evidence of DENV C binding core histones in the nucleus and death domain associated protein (DAXX) [59,60]. These interactions can hinder nucleosome remodeling or prevent binding to necessary transcription factors, respectively, ultimately perturbing gene expression. Although these and other studies have shed some light on alternative functions of flavivirus capsid, capsid has been shown to interact with many host proteins [61]. An in-depth look into the many pathways that are potentially affected by these interactions will provide greater understanding of flavivirus pathogenesis and the roles that capsid plays.

## 4. Indiscriminant Binding of Flavivirus Capsid to Nucleic Acids

Based on the very basic surface of capsid proteins it is reasonable to consider that capsid binds to the negatively charged phosphate backbone in all nucleic acids. In 2018, Shang et al. [32] performed an isothermal titration calorimetry assay to measure the binding affinity of ZIKV C to four types of nucleic acid: 5′ UTR of the ZIKV genome (ssRNA), dsRNA, ssDNA, and dsDNA, which may be found in the nucleus. Interestingly their studies indicated that ZIKV C bound all nucleic acids with affinities in the nanomolar range. This high affinity for all nucleic acids lends itself to the dsRNA binding ability which prevents dicer activity in mosquitos, as noted previously [48], and others have shown that ZIKV capsid’s ability to bind ssDNA is made possible by the positively charged surface of the protein [49]. Although the affinity for all nucleic acids is high, the specificity appears to be low. The dissociation constant for DENV capsid is roughly 20 nM [15,17]. Considering the wide range of nucleic acid binding and the varied localization of flavivirus capsid it is reasonable that there may be additional (potentially transient) nucleic acid interactions for the capsid proteins that have yet to be described.

## 5. Flavivirus Capsid as a Target for Antiviral Treatment

Although in theory any viral protein may serve as an antiviral target, flavivirus capsid has many structural features and functions that can steer antiviral drug design. Previously, the E, NS3, NS5, and NS4B flaviviral proteins have served as targets, but therapeutic candidates have fallen short due to undesirable pharmacokinetics, poor selectivity, permeability or stability [54]. Since capsid proteins have served as valid targets for antivirals in many other virus families, including alphaviruses and retroviruses [62,63,64,65,66,67], they may serve as a valuable target in the case of flaviviruses. Capsid has no enzymatic core, therefore anti-viral strategies would focus on disrupting capsid interactions with other host or viral factors. There are three important interactions of interest: capsid-protein interactions (homo-dimer formation, formation of the nucleocapsid, or interactions with host proteins), capsid-RNA interactions, and capsid-phospholipid membrane interactions.

Capsid self-interactions, such as dimerization and nucleocapsid formation, aid in the packaging of the viral genome. Hindering these processes would result in viral attenuation. Conversely, stabilizing capsid dimers may prevent genome release upon cell entry. One example is the small molecule inhibitor of DENV C, ST-148 [68,69]. This small molecule binds in the hydrophobic pocket between two capsid dimers, stabilizing this interaction. This has been shown to reduce the number of virus particles produced/released as well as prevent genome release [70]. Other capsid interactions, such as those with host proteins are also valuable targets (see Table 1). DENV capsid binds nucleolin, as noted previously, disrupting ribosome biogenesis. siRNA treatment with aptamer AS1411 prevents nucleolin’s interaction with capsid proteins by directly binding to nucleolin precluding capsid interaction [56]. Therefore, targeting host factors rather than viral proteins is an attractive strategy to avoid virus adaption and the evolution of drug resistance. Although aptamers designed to bind capsid may prevent a number of its various interactions.

Little work has been done to provide greater understanding of how nucleic acids, in particular how RNA species, may compete for capsid binding. Although we have seen flavivirus capsid’s ability to essentially bind any nucleic acid, it is not clear if capsid has a preference for binding RNA motifs or secondary structures. Recently, Boon et al. showed that capsid’s interactions with viral genome indicate some preference for G-quadruplexes *in vitro* [71]. It has also been shown that capsid proteins have the ability to anneal RNAs, indicating the ability to interact with both ss- and dsRNA [34], in contrast to predictions that capsid would specifically bind single-stranded RNA [10]. However, it is entirely possible that capsid binds both ss- and dsRNA in the process of compacting the genome. Therefore, a comparison of what nucleic acids capsid binds *in vivo* is necessary. Understanding how nucleic acids interact with capsid is necessary to determine if an RNA oligo could be used to compete with the viral genome for capsid binding. It may be possible that an aptamer may also prevent capsid’s interactions with host proteins as indicated above.

Many have already begun looking into the use of small molecules or peptides to prevent the association of flavivirus capsids with membranes [54]. Nordihydroguaiaretic acid (NGDA) is a small molecule that reduces the amount of lipids in the cell by causing an increase in certain effector molecules. NGDA prevents lipogenesis via its impact on the effector AMPK-α and increases fatty acid oxidation through PPAR-α [72]. Similar effects were shown with fatty acid synthase inhibitor C75 [17]. Although these small molecules do not bind capsid protein, they effectively serve as antivirals as capsid can no longer associate with membranes that are not present. Inhibitors of cholesterol transporters such as ABCG1 have also shown promise as effective flavivirus antivirals [39]. In 2012, Martins et al. investigated DENV capsid interactions to lipid droplets by producing peptides mimicking the N-terminal domain of the capsid. In further testing they discovered that treatment with one of these peptides, pep14-23, not only bound lipid droplets but prevented full length capsid from interacting with the membrane [14]. Thus, antivirals aimed at preventing capsid’s interaction with lipid droplets may gain more traction in the future.

## 6. Considering Capsid in Flavivirus Vaccine Design

Considering the potential symptoms of hemorrhagic fever, encephalitis or even death and the geographic expansion of mosquito vectors, established and emerging flaviviruses are an increasing public health risk globally [37,41,73,74]. There is a clear need for safer and more efficacious flavivirus vaccines. One success story is that of the veterinary vaccine, RecombiTEK (Merial) available since 2004, used to vaccinate horses against West Nile virus. This recombinant virus uses canarypox as a vector to express WNV E and prM proteins. Interestingly there are multiple equine WNV vaccines commercially available: RecombiTEK, a live-attenuated vaccine, and a chimeric vaccine containing the 17D backbone but expressing WNV E and prM [75]. All of these allow the survival of all horses challenged with WNV infection, compared to the devastating ~30% fatality rate seen during the breakout in the United States in 1999 [76]. There are currently only a few flavivirus vaccines commercially available to humans. The two most notable are the live-attenuated YFV 17D vaccine and *Dengvaxia* (a tetravalent DENV chimeric vaccine), but there are also vaccines available for JEV [7,8,77,78]. Although the 17D vaccine was generated in the 1930s, it is still widely used to immunize people today as it is one of the safest and most effective vaccines available. With six countries producing three strains of this live-attenuated vaccine, the World Health Organization (WHO) Fhas set a course to eliminate yellow fever epidemics over the course of a decade [79]. Although this vaccine is widely used, it does however have a its share of adverse effects [80]. Mild symptoms are seen in 25% of immunized individuals and 1 in 55,000 experience a severe allergic reaction. Symptoms can extend to severe nervous system (1 in 125,000) or even extensive organ failure (1 in 250,000) with over half of those entering organ failure passing away [81]. Even with these statistics, the effective use of 17D as vaccine garners hope for the development of other flavivirus vaccines. The chimeric vaccine available for DENV, *Dengvaxia,* uses 17D as a template or backbone but substitutes the genes for the E and prM proteins from the different DENV serotypes. It is a tetravalent vaccine, but has only been shown to confer immunity for two of the four serotypes of DENV. Studies have shown that administration of this vaccine resulted in multiple cases of serious injury to children in the Philippines [8,9]. As mutating or deleting capsid hinders the production of infectious particles, a more serious look at how capsid is involved in packaging and how we can use that information to develop better vaccines is warranted. Here, we will focus on how capsid can be taken into consideration for vaccine design and development, particularly in live-attenuated, DNA, and subunit vaccines.

Traditionally, live attenuated viruses were generated by serial passaging of virus in cell culture or animal tissue until virulence greatly decreased [77,82]. The YFV vaccine noted above, 17D, was generated roughly 80 years ago via serial passaging of the wild-type Asibi strain 176 times in mouse and chicken tissues [77,82]. The primary method of attenuation in 17D is its reduced genetic diversity within the YFV quasi-species, indicating high fidelity caused by mutations within nonstructural genes [82]. Interestingly, the majority of mutations present in 17D are silent, in that they code for the same amino acid. Therefore, it is possible that codon de-optimization aids in attenuation. However, E2992G in the NS5 (RdRP and methyl-transferase) may provide the increased fidelity of the polymerase [83] and mutations in the envelope protein may also hinder receptor binding or membrane fusion [77]. This vaccine has many substitutions throughout the genome, primarily in non-structural genes. Interestingly however, F49G in the capsid is generally overlooked. This is the only mutation in the capsid gene [83], indicating that perhaps capsid must be conserved to package the genome, allowing multiple rounds of infection. The substitution of a bulky side chain for a single hydrogen may impact interactions with lipid membranes and could be worth exploring.

**Figure 3:**
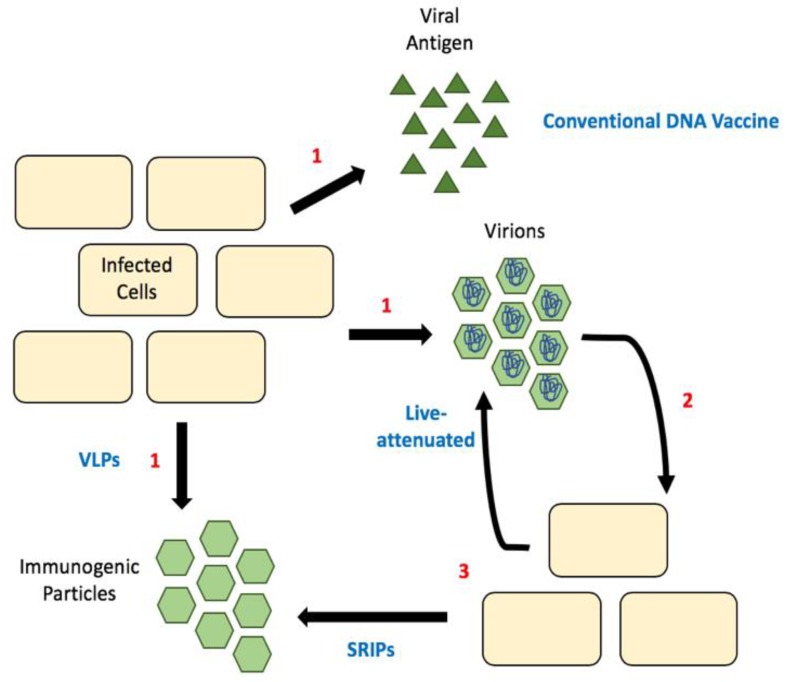
Comparison of different vaccine strategies. (**1**) Release of antigenic material, which can be viral proteins, empty particles (SVPs) or infectious particles. (**2**) Released infectious particles can undergo the first round of replication, (**3**) this produces either more infectious particles (as is the case with live-attenuated vaccines) or empty particles called SRIPs. Sub-viral particles (SVPs); single-round infectious particles (SRIPs).

Luckily, reverse genetics lets us decipher what effect different mutations and deletions have on different viral processes. For example, it is now known that mutations in the 3′ UTR result in hinder flaviviral replication [84]. Unsurprisingly, many mutations to the gene encoding capsid protein result in the production of sub-viral particles (SVPs, Figure 3) [85]. These SVPs are immunogenic as they display the viral surface proteins E and M, but non-infectious because they fail to package the viral genome. Thus, they have the potential be used as vaccines. The first flavivirus this was observed in was tick-borne encephalitis virus [85]. Since then we have seen the production of these attenuated viral genomes for many flaviviruses, including WNV, ZIKV and DENV [86,87,88]. These are initially produced in cells expressing wild-type/full-length capsid and the virions produced can be used for immunization. In mice, such vaccines have been shown to confer immunity with as little as one dose and protect fetuses of pregnant females [86]. To date, there has not been a strong push for capsid mutant live-attenuated vaccines in clinical trials, although their efficacy in animal models seems promising.

Additionally, a number of flavivirus DNA vaccines have entered clinical trials, but they primarily focus on the expression of envelope and membrane proteins to produce SVPs, or recombinant viruses [89,90,91,92]. Similar to the live-attenuated vaccines above, a DNA vaccine with mutations in capsid protein would produce SVPs. One concern is that it may require a lot of DNA to immunize even a single person. This highlights the potential for single-round infectious particles (SRIPs) in flavivirus vaccine development [89,93,94]. SRIPs are DNA vaccines that are comprised of a plasmid encoding for the viral genome lacking the capsid and with capsid provide via trans-expression. These allow for the production of authentic infectious particles within the first infected cell, but packaging viral genomes that are defective, as they lack the capsid gene. These defective particles are used as the vaccine, resulting in the production of immunogenic SVPs in the immunized individual (Figure 3). This greatly increases the number of SVPs produced and thus provides a bump in immunogenic particles resulting in a greater immune response [94]. These vaccines are particularly promising as the majority of particles produced would be non-infectious but should elicit an immune response sufficient for immunity.

One potential issue with the use of these DNA vaccines, or even capsid mutant live attenuated vaccines, is the amount of SVPs that would need to be produced to confer immunity, and of course there is also the concern for antibody-dependent enhancement (ADE). ADE occurs when antibodies circulating from a previous infection bind new antigen without neutralizing virus, resulting in a more efficient uptake into monocytes. This is common across the dengue serotypes and extends to Zika virus as well [95,96]. Therefore, there may also be promise in a subunit vaccine containing a viral antigen, such as soluble capsid protein. This has been observed in immunization of ducks for Duck Tembusu virus, a new member of the *Flavivirus* genus [97]. Although the capsid protein is not exposed on the surface of flaviviral particles, the protein may elicit both an adaptive and innate immune response. This is interesting because we typically consider responses to the surface proteins E and M. It seems plausible that flavivirus infected cells may lyse, releasing capsid or even present fragments of capsid via the up-regulated MHC I pathway—resulting in these same responses in infected individuals [98,99,100,101]. Without crucial studies looking at the effectiveness of such a vaccine and its safety in humans we cannot comment further, but this may be an interesting path worth investigating.

With climate change broadening the range of mosquito vectors and the potential severity of symptoms associated with flavivirus infections, these emerging viruses are becoming a global issue. Flaviviruses are already the most prevalent viral infections in the world, with almost 400 million cases per year, roughly a quarter of those symptomatic, and resulting in 25,000 deaths per year [102]. As noted previously, the commercially available vaccines leave much to be desired and there are many flaviviruses that do not currently have a vaccine [9,77,80]. It appears that changing the focus from the surface proteins (E and M) to capsid would be substantiated. There is certainly potential in designing vaccine with capsid mutations or deletions or even providing a subunit vaccine containing the capsid protein.

## 7. Conclusions

When placed into the context of the viral life cycle it is interesting to consider the various interactions of flavivirus capsid proteins. Antivirals such as ST-148 have been investigated for having a stabilizing effect, preventing uncoating and genome release of DENV upon viral entry [69,70]. Of course, the canonical function of capsid is to package the viral genome so that it may be successfully transmitted to another host cell. This has been the focus of vaccine development, in the production of assembly or packaging defective viruses. Before capsid can form the nucleocapsid, it is stored on lipid droplets via interactions with host proteins MBOAT2, AUP1 and others [14,15,16,17,61]. In order for capsid dimers to make it to and from the lipid droplets, interactions with vesicular trafficking proteins is necessary (YKT6, USE1, etc.) [61]. Once enough protein has been translated and viral genomes replicated capsid is shuttled back to the ER for nucleocapsid assembly, again utilizing host vesicular transport machinery. This would appear to be the end of the capsid protein’s role in the flavivirus life cycle, however, it is now clear that there are a number of other roles and interactions that capsid proteins have that aid in the pathogenesis of these viruses, as highlighted in Table 1. For example, capsid’s ability to enter the host nucleolus resulting in ribosomal stress and Tp53-mediated apoptosis induction in neural progenitors is a concern for development of microcephaly in fetuses growing within infected mothers [20,73].

The current understanding of flavivirus capsid and its role in the virus life cycle is based on information from the capsid structures [35,42], information about nucleocapsid assembly [15,103,104], and the protein’s interactions with host proteins with hopes of potential discovery of drug targets [54,61,69,72]. Upon cleavage from the viral polyprotein by signal peptidase, capsid can interact with vesicular transport proteins to move towards storage on lipid droplets or towards the nucleus [61]. Capsid dimers interact with the membrane of lipid droplets, aiding in both viral packaging and storage of capsid prior to packaging [14,17,35]. We speculate that there may be an equilibrium between capsid dimers stored on lipid droplets for particle formation and capsid dimers able or available to enter the host nucleus and nucleolus. It has been shown that flavivirus infections can alter the host transcriptome including up-regulation of lipid synthesis [38,40]. Perhaps the equilibrium noted above could be influenced by the number and size of available lipid droplets within a host cell, i.e., a cell’s capsid storage capacity. Flavivirus capsid apparently binds nucleic acids non-specifically [15,48,54,57] but capsid is not the only soluble viral protein with the ability to bind double stranded nucleic acids [27,105]. Many transcriptome studies have been done in the context of these viral infections, however it is unclear if capsid or other soluble viral proteins, such as NS5 which has been shown to enter the host nucleus, are responsible for transcriptome-wide changes. Studies on transcriptome changes in response to the soluble viral proteins individually may shed light on specific changes in the host transcriptome. In addition, although various host proteins have been found interacting with the capsid protein [61] only a few of these interactions have been thoroughly studied and although some drugs have been designed to target these interactions there is still no treatment for these infections.

In the future we expect to see more studies investigating the implications of the various interactions and considering how disrupting they are, which may prove helpful in the design of novel antiviral treatments. Particular consideration should be taken to review all pathways that could potentially be affected by capsid interactions based on the currently identified protein interactions. In addition, interactions resulting in clear pro- or anti-viral processes are prime targets for anti-viral design. It is also pertinent to garner better understanding of how capsid interacts with viral genome and other nucleic acids in host cells. With immunoprecipitation pull down of nucleic acids bound to capsid and next generation sequencing it may be possible to elucidate binding patterns, whether they are motifs or RNA structural features. Overall, there is much to be desired in terms of the field’s knowledge pertaining to what specific interactions flavivirus capsid proteins make within cells and how those interactions are relevant in both pathogenesis and drug or design.

## Figures and Tables

**Table 1:** Host proteins that interact with capsid and how antivirals preventing this interaction may affect the virus.

Location.	Pathway	Protein	Result	Pro- or Anti- viral	Antiviral treatment could	References
Endoplasmic Reticulum	Vesicular Transport	YKT6 SCFD1 USE1 VAPB STX8 TMEM85 SAR1B	Movement of capsid from site of cleavage to lipid droplets or nucleus	Pro	Prevent capsid storage on lipid droplets, entry into nucleus/nucleolus, prevent particle assembly	[61]
	Signal Peptidase Complex	SPC52 SEC11A SPC53	Cleavage of capsid from flavivirus polyprotein			
	ER Stress Response	PARP16 DORGK1 UBXN8 RTN3 SLC33A1	Cell survival or cell death	Anti		
Nucleus	Importins	IPO7 IPO9	Targeting of viral capsid to nucleus	Both	Prevent capsid interaction & entry into host nucleus	[19,20,30,36,61]
	LINC Complex	SUN1 SYNE2	Interaction with nuclear membrane			[61]
	NPC	NUP35 NUPL2 TMEM48 POM121	Entry of viral capsid into host nucleus			[61]
	NMD Pathway	PABPC UPF1	Prevent degradation of viral transcripts, increase degradation of host transcripts	Pro	Allow degradation of viral transcripts by host cells	[58]
	Host Transcription	DAXX	Prevent binding of host transcription factors		Prevent changes in host gene transcription	[60]
		Core Histones	Change position of histones			[59]
Nucleolus	p53 regulation	MDM2	p53 induced apoptosis	Anti		[23]
	RNA methylation	NSUN2	Modified methylation of tRNAs, mRNAs, and ncRNAs	Not yet understood	Prevent changes in transcript methylation	[61]
	Ribosome biogenesis	NOL8 NOP16 NOC4L B23 NPM1 nucleolin	Ribosomal stress	Anti		[61]
						[21]
						[56]
	Protein turnover	Jab1	Remove viral capsid protein			[19]
Lipid Metabolism	Lipid droplets	AGPAT6 LPCAT2 AUP1 MBOAT2	Storage of accumulated capsid until particle assembly	Pro	Cause issues with particle formation; formation of VLPS	[61]
	Sphingolipid Metabolism	KDSR TEX2	Initial budding into ER			
	Lipid binding	GRAMD1A GRAMD3	Interaction with lipid membrane			
	Ceramide Metabolism	SMPD4 CERS2 GBA2	Initial budding into ER			

Notes: The table is color coded for easier viewing: ER: blue, nucleus: green, nucleolus: red, and lipid metabolism: orange.
